# A seasonal study of a passive abandoned coalmine drainage remediation system reveals three distinct zones of contaminant levels and microbial communities

**DOI:** 10.1002/mbo3.585

**Published:** 2018-04-25

**Authors:** Michelle M. Valkanas, Nancy J. Trun

**Affiliations:** ^1^ Department Biological Sciences Duquesne University Pittsburgh PA USA

**Keywords:** abandoned mine drainage, bacterial communities, passive remediation

## Abstract

A passive remediation system that treats coalmine drainage was sampled to determine the impact seasonal changes had on water quality and microbial diversity. Every quarter for 1 year, water‐soil slurries were collected at the influent of the 5 settling ponds and the wetlands, and the effluent of the system. The concentration of 12 metals and sulfate, as well as sequences from the V4 region of the bacterial 16S *rrn* genes were determined. The water quality analysis revealed high levels of iron and sulfate, and measurable levels of Al, Ba, Cu, Pb, Mn, Sr, and Zn. Iron increased 25‐fold in the summer and spikes in metal concentrations were observed during several seasons in pond 3 and the wetlands. These spikes cannot be explained by abiotic chemical reactions in the neutral pH found in the pond. Based on contaminant levels and microbial community composition, our results indicate that there were 3 unique environments in the system (ponds 1 and 2; pond 3; pond 4 through the end) and that changes in contaminant levels and bacterial composition in these environments correlated with seasonal variation. Iron and sulfate are the most prevalent contaminants in the system. An examination of sequences from known iron‐ and sulfur‐cycling bacteria demonstrated that there were more iron‐reducing (IRB) bacterial sequences than iron‐oxidizing (IOB) (137,912 IRB vs. 98,138 IOB), the two groups of bacteria were found mainly in the fall and winter samples, and were prevalent in different ponds. There were more sulfur/sulfide‐oxidizing (SOB) bacterial sequences than sulfur/sulfate‐reducing (SRB) bacterial sequences (72,978 SOB vs 30,504 SRB), they were found mainly in the fall and winter samples, and the sequences were mixed in ponds 4, 5 and the wetlands effluent. Iron is remediated in this system but sulfate is not.

## 1 INTRODUCTION

Abandoned coalmine drainage (AMD) is a significant contributor to contamination in watersheds in many countries, and is the leading cause of contamination in Pennsylvania (PA). It impacts over 3,000 miles of streams in PA (Cravotta, 2010). One third of all coal mined in the United States before 1977 came from PA, and over 11,000 abandoned mines have been documented in the state (PA‐DEP, 1998; PSU, 2017). AMD from coal mines impacts agriculture, drinking water sources, and aquatic habitats (Hallberg, 2010). It is generally high in dissolved iron, aluminum, manganese and sulfate and can contain trace heavy metals (Hedin, Narin, & Kleinmann, 1994). It has been estimated that it will cost up to $15 billion to restore PA watersheds that have been damaged by AMD (Cravotta, 2010).

Passive AMD treatment systems are being constructed at many abandoned mine sites, with approximately 300 remediation systems currently in operation in PA (for a description of the systems, see http://www2.datashed.org). This represents the highest number of passive remediation systems in any state. These systems are designed in a variety of ways, however, they all aerate the AMD at the beginning of the system, have contaminant settling ponds, and often follow the settling ponds with constructed wetlands (Hedin, Weaver, Wolfe, & Watzlaf, 2013; Hedin et al., 1994; Turner & McCoy, 1990; Watzlaf, Schroeder, & Kairies, 2000; Zipper, Skousen, & Jage, 2011). Aerating AMD results in iron oxidation, followed by precipitation of the iron oxide particles. Each settling pond is deeper than the settling pond immediately following it so that the precipitated iron is trapped and retained in the pond as sediment. Acidic AMD requires elevation of the pH prior to aeration (Hedin, Watzlaf, & Nairn, 1994; Turner & McCoy, 1990), while alkaline AMD does not. pH adjustment is necessary because geochemical precipitation of metals is faster at pH 6–8, especially for iron, the most prevalent contaminant in AMD (Hedin, 2008). Approximately half of the AMD in PA is acidic (pH < 5) and half is alkaline (Cravotta, 2010). Passive systems are a useful choice in remediation efforts because they are cost effective, can be implemented in geographically remote areas and allow removal of contamination through gravitational, geochemical, and biological processes (Hedin et al., 2013; Zipper et al., 2011). From a geochemical perspective, passive systems have proven to be effective in increasing pH and removing heavy metals (Hedin, 2008; Hedin, Weaver, Wolfe, & Weaver, 2010; Hedin et al., 1994, 2013; Kairies, Capo, & Watzlaf, 2005).

Several studies have confirmed a strong microbial influence in passive remediation systems from both acidic and alkaline AMD (Bier, Voss, & Bernhardt, 2015; Jones et al., 2015; Senko, Wanjugi, Lucas, Bruns, & Burgos, 2008; Whitney, Tracey, & Jeanne, 2013). Bacteria can remove metals through adsorption to a surface, biosorption to biomass, immobilization, and precipitation, as has been successfully demonstrated in bioreactors (Chang, Hsu, Chiang, & Su, 2006; Diels et al., 1995; Scott & Karanjkar, 1998; Scott, Karanjkar, & Rowe, 1995; Travieso et al., 2002). They can also resolubilize metal precipitates, increasing soluble contaminant levels. It has been demonstrated that manganese and iron oxide precipitates trap heavy metals and resolubilization of these precipitates releases the trapped metals into solution (Bourg & Loch, 1995; Jenne, 1968; Shope, Xie, & Gammons, 2006). The bacterial community composition between acidic and alkaline environments exhibits different phylogenetic prevalences. For example, *Acidithiobacillus ferrooxidans* and *Leptospirillum ferrooxidans,* the major iron‐oxidizing bacteria in acidic AMD, have an optimal pH for growth of pH < 4 and are not usually found in alkaline systems (Baker & Banfield, 2003; Johnson & Hallberg, 2003). Despite compositional differences, it has been shown that there are biological communities present in both types of systems that are capable of both positively and negatively influencing remediation (Bier et al., 2015; Senko et al., 2008).

We examined the seasonal changes in water chemistry and microbial communities at Wingfield Pines, a circumneutral, passive bituminous coal mine remediation system located in Allegheny County, southwest of Pittsburgh, PA. Constructed in 2009, Wingfield Pines collects AMD from several mineshafts and gravity feeds it to the start of the remediation system. The AMD flows through a pipe containing 2–5 cm holes to create a sprinkler‐like effect and aerate the AMD, allowing for the initial precipitation of iron and other metals in pond 1. The AMD then flows through four successive settling ponds (ponds 2, 3, 4 and 5) to allow for additional oxidation and precipitation of iron, followed by a constructed wetland before emerging into a small stream that flows back into Chartier's Creek. In this study, we examined the efficiency of contaminant removal over the course of four seasons and determined the composition of the bacterial communities at the entrance to each pond during each season. Our data indicate that 81% of the iron was removed at the winter, spring and summer time points but only 51% was removed at the fall time point. Additionally, pond 3 and the wetlands exhibited spikes in certain metals at neutral pH, contrary to chemical data indicating that these metals should only be soluble in acidic conditions. Sulfate is not remediated in this system. Metagenomic analysis indicated that the beginning of the system had the most variability in relative abundance of different bacterial sequences over the four seasons, while the end of the system exhibited relatively stable bacterial communities. An analysis of the bacterial sequences found in the system and known metabolic pathways present in these bacteria provide testable hypotheses for the effects of bacteria on AMD remediation in Wingfield Pines.

## 2 EXPERIMENTAL PROCEDURES

### 2.1 Sample collection

**Figure 1:**
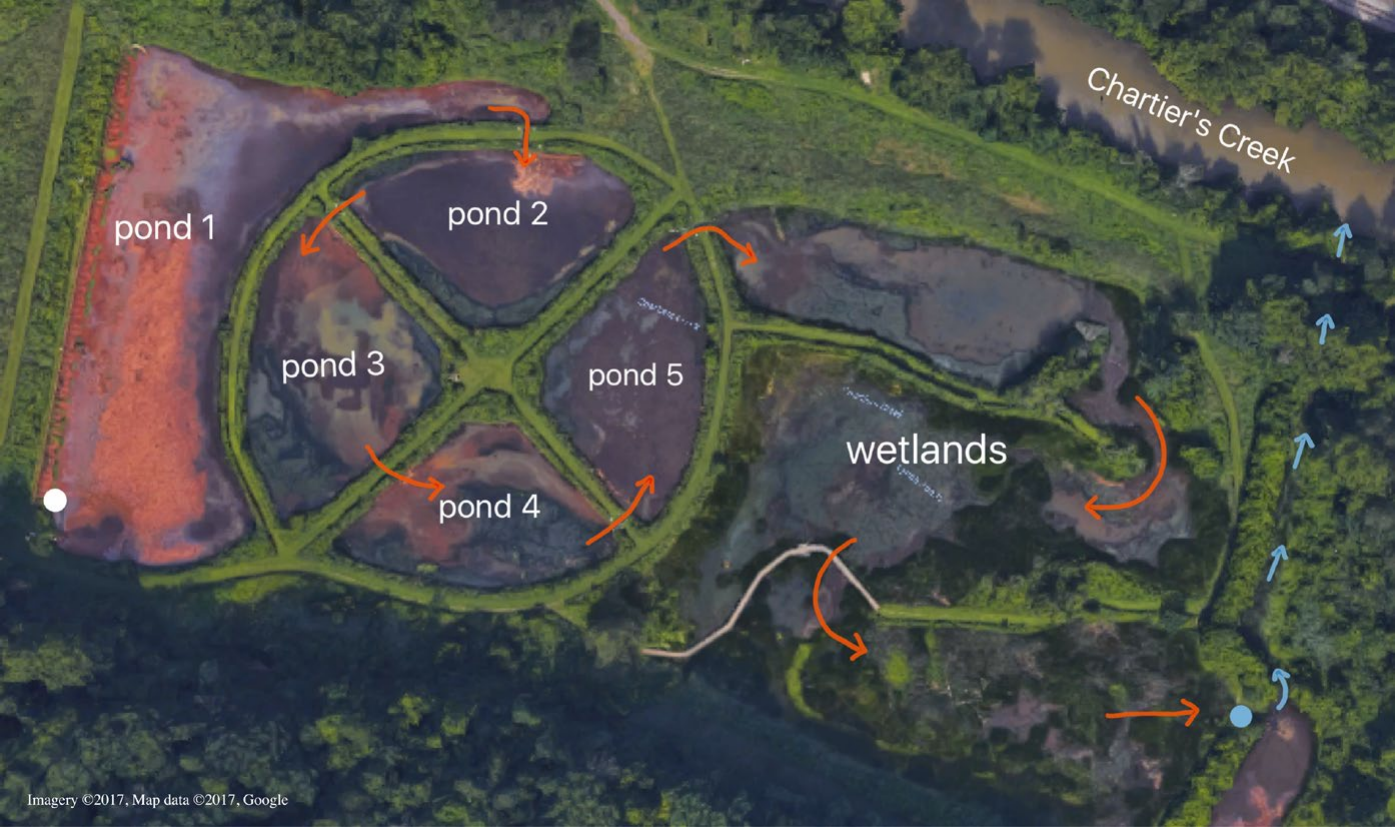
Wingfield Pines passive abandoned coalmine drainage remediation system, Upper St. Clair Township, southwestern PA. The mineshafts were capped underground and the AMD water is gravity fed to the start of the system (white dot in pond 1). Water flows through a large pipe containing equally spaced holes along the entire left side of pond 1. Water flows through the system in the direction indicated by the orange arrows. The effluent from the system is indicated by the blue dot. Blue arrows indicate the path of the remediated water to Chartier's Creek. The system was designed with a flow rate of 1,500–2,000 gallons/min

Wingfield Pines is a passive bituminous coal mine remediation system that discharges an alkaline water flow. The remediation system consists of a gravity fed underground pipe from the mine shafts to an aeration pipe that releases the discharge into settling ponds (Figure 1). There are 5 settling ponds, followed by a constructed wetland. The water is discharged into a small stream that empties back into Chartier's Creek. Sampling was performed quarterly on April 30, 2015, July 20, 2015, October 28, 2015, and January 27, 2016 to achieve a seasonal representation of the system. NOAA reported an average high temperature of 17.2°C, average low temperature of 5.6°C and 10.8 cm of precipitation (rain and snow) for April 2015. July 2015 had an average high of 28.3°C, average low of 17.8°C and 9.2 cm precipitation. October 2015 had an average high of 17.2°C, average low of 7.2°C and 8.5 cm precipitation. January 2016 had an average high of 1.7°C, average low of −7.8°C and 41.6 cm of precipitation (http://www.ncdc.noaa.gov/cdo-web/datasets). Due to the depth the AMD is coming from, the remediation system never completely freezes. Seven samples were collected for each time point, one at the influent of each settling pond (ponds 1–5), one from the influent into the wetlands and one from the effluent out of the wetlands. The influent to pond 1 is the influent into the remediation system and the effluent from the wetlands is the effluent out of the remediation system. Fifty milliliters of mixed slurry were collected for each sample, consisting of approximately half water and half soil.

### 2.2 Water chemistry analysis

The samples were transported at ambient temperature to the laboratory and the pH was measured immediately. A 50‐ml sample was centrifuged at 850*g* for 20 min. The supernatant was used for water chemistry analyses and the pellets were stored at −80°C prior to DNA extraction. Water samples were transported on the day of their collection to R.J. Lee Group, Inc. (Monroeville, PA, http://www.rjlg.com) for water quality analysis. Water was tested for total metals (Al, As, Ba, Cd, Cu, Fe, Mn, Ni, Pb, Se, Sr, and Zn) by inductively coupled plasma‐atomic emission spectrometry (ICP‐AES) per EPA 200.7‐PA and sulfate analysis using ion chromatography (IC) per EPA 300.0‐PA.

### 2.3 DNA extraction and 16S rRNA gene sequencing

Samples containing water and soil mixtures were centrifuged to pellet the bacteria. Total DNA was isolated from 0.25 g of the pellet from the 28 samples, using a Powersoil Kit (MoBio Laboratories Inc., Carlsbad, CA). A control consisted of sterile nanopure water extracted in place of soil. The V4 region of the 16s *rrn* genes from bacterial DNAs were amplified by Illumina tag (itag) PCR using the 515F forward primer and the Illumina 806R reverse barcoded primer (16S Illumina Amplicon Protocol, http://www.earthmicrobiome.org). Individual barcoded samples were prepared and sequenced (Ulrich et al., 2016) by Wright Labs (Huntingdon, PA).

A control of extracted sterile nanopure water has been reported to show contamination in 16S *rrn* gene sequencing experiments (Salter et al., 2014). Our control contained 4,268 sequences and 84 species, most of which were in trace amounts (<1%). Five groups account for 69% of the 4,268 sequences: Acidobacteria CCU21 (17%), Chloroflexi Ellin6529 (15%), Delta‐proteobacteria Desulfobacteraceae (11%), Deltaproteobacteria Desulfobulbaceae (17%), and Verrucomicrobiaceae (9%). Of these, only Desulfobacteraceae and Desulfobulbaceae (unspecified) were found in the remediation site samples in high abundance as well. For this reason, they are not considered significant in the analyses of the site samples. The other three species are found in trace (<1%) amounts in the Wingfield Pines samples, except in three occurrences where they are below <3% of the sequences in the samples.

### 2.4 Statistical analyses

The sequences were trimmed to 252 bp (average sequence length was 151 bp) and quality filtered with an expected error rate of less than 1% (USEARCH v7, (Edgar, 2010)). Chimeras were removed using USEARCH (Edgar, 2010). The sequences were analyzed using the software package QIIME‐1.9.0 (Caporaso et al., 2010, 2011). After quality filtering, the 29 samples (28 samples and a control) yielded 2,420,962 paired reads. All samples contained >2,000 sequences. Using the USEARCH algorithm, open reference operational taxonomic units (OTUs) were selected and assigned taxonomy using the Greengenes 16S *rrn* gene database and a 97% identity match (DeSantis et al., 2006). Using Qiime‐1.9.0, a Biological Observation Matrix (biom) table was constructed of the OTUs with their assigned taxonomy, and the biom table was the core of all downstream analyses.

Using the python script summarize_taxa.py in Qiime‐1.9.0, relative abundance was generated. Bar graphs were created with all phyla that account for at least 1% of the samples, and the rest of the phyla were placed in an “other” category. The three Archaea phyla (Crenarchaeota, Euryarchaeota, and Parvarchaeota*)* were plotted as a group. The chloroplast sequences, containing eight order (Chlorophyta, Crytophyta, Euglenozoa, Haptophyceae, Rhodophyta, Stramenopiles, Streptophyta, UA01), were grouped together as well. The Archaea and chloroplast groups were separated out even though they did not follow the 1% rule, as applied to the bacterial relative abundance groups. Based on abundances >1%, Proteobacteria were divided into classes and plotted to show the relative abundance of the 5 major Proteobacteria classes. Any unidentified classes of Proteobacteria as well as Zetaproteobacteria were grouped into the other category.

Qiime‐1.9.0 was used to generate alpha‐diversity multiple rarefactions plots. Sequences accounting for <0.005% of samples were removed before rarifying OTU tables. Summer site 1 had <1,000 samples when <0.005% sequences were removed and no conclusions about summer site 1 richness could be determined. All samples were rarified, assuring an even sequencing depth with a minimum of 100 sequences and a maximum of 6,000 sequences, using a step size of 500 sequences per sample for 20 iterations. Using phylogenetic distance (PD) whole tree, Heip's evenness, Chao1, and observed species richness metrics, alpha rarefactions were collated.

Using weighted UniFrac distances, beta diversity was characterized between site samples (*n* = 28), as well as with the control (*n* = 29). Principal‐coordinate analysis (PCoA) plots were created with Qiime‐1.9.0, using a cumulative sum scaling (CSS) normalized OTU biom table of unrarefied OTUs to visualize seasonal and site changes in bacterial community composition (Lozupone & Knight, 2005). PCoA plots were visualized using EMPeror (Vázquez‐Baeza, Pirrung, Gonzalez, & Knight, 2013). To determine the significance of variation explained by season and site location, Adonis tests were performed on weighted UniFrac values. Adonis returns a *p* value for significance and an *r* ^*2*^ value indicative of the amount of variation attributable to the selected variable. An α value of 0.05 was considered significant.

## 3 RESULTS

### 3.1 Sampling location

The Wingfield Pines passive AMD remediation system (Figure 1) is in southwestern Pennsylvania at a latitude 40° 20′ 26.9988″ N and a longitude 80° 6′ 34.9992″ W. Construction was completed in 2009, and the bacterial communities accumulated on their own, without deliberate input. The discharge from Wingfield Pines empties into Chartier's Creek, which is part of the Ohio River watershed. The water in Wingfield Pines has an average pH of 7 with high levels of iron, and elevated levels of a variety of other heavy metals and sulfate (Table S1).

### 3.2 Water quality analysis of seasonally collected samples

Twenty‐eight samples were collected over a period of 1 year. One sample was collected at the influent of all 5 settling ponds (pond 1–5) and the influent and effluent of the wetlands (Inf Wetlands and Eff Wetlands). The influent of pond 1 is the influent into the remediation system, and the effluent of the wetlands serves as the effluent from the system. (Table S1) shows the yearly average for the contaminants tested. Copper, lead, and nickel concentrations remained under detection limits (<0.01, <0.03, <0.01 mg/L) through most of the remediation system, except for pond 3, pond 4, and the effluent, where each metal could be detected. The pH values were neutral and consistent throughout the system, with a slight increase from the influent (pH 6.89) to the effluent (pH 7.25).

**Figure 2:**
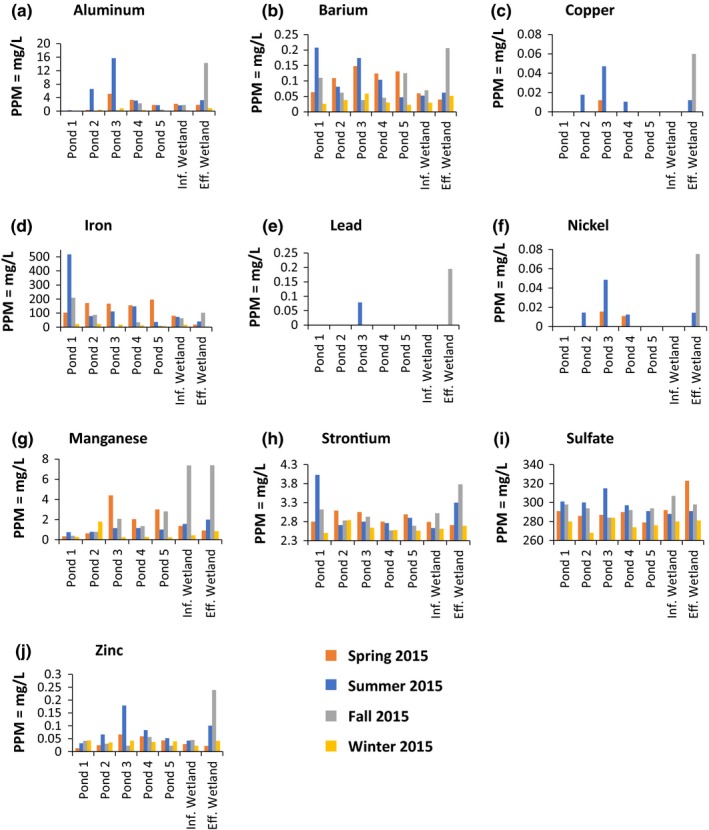
The water quality across the remediation system by season. Graphs for arsenic, cadmium, and selenium were not included because all the values were under the detection limit. Panel A contains aluminum concentrations by pond and season. Panel B shows barium levels; Panel C, copper; Panel D, iron; Panel E, lead; Panel F, nickel; Panel G, manganese; Panel H, strontium; and Panel I, sulfate

When water quality was analyzed by season, several trends were uncovered (Figure 2). The influent to the system contained the highest amount of total iron in the summer (518 mg/L; Figure 2D), more than double the next highest season (fall = 209 mg/L). The total iron in the effluent from the system was the highest in the fall (102 mg/L). The system removed the most iron in 3 out of the 4 seasons (81%), but was less effective in the fall, where only 51% of the iron was removed.

Aluminum, copper, lead, nickel, and zinc were mainly found in low levels throughout the system but showed defined spikes in pond 3 in the summer and the system effluent in the fall (Figure 2, panels A, C, E, F, and J, respectively). The average amount of manganese increased as the AMD worked its way through the system, showing spikes in pond 3 in the spring (4.4 mg/L), the influent of the wetland (7.38 mg/L) in the fall, and the system effluent in the fall (7.39 mg/L; Figure 2G). Barium levels, while generally low, varied seasonally throughout the remediation system with the concentration never elevated above ~0.2 mg/L (Figure 2B). Strontium levels showed minor fluctuations seasonally with pond 1 having the highest and lowest levels in summer (4.03 mg/L) and winter (2.5 mg/L), respectively (Figure 2H). All samples were tested for arsenic, cadmium, and selenium (not shown) and results were always under the calibrated detection limit. Sulfate levels fluctuated slightly throughout the system during the year, but the influent and effluent remained consistent (±3 mg/L; Figure 2I).

### 3.3 Analysis of bacterial communities using 16S *rrn* sequencing data

**Figure 3:**
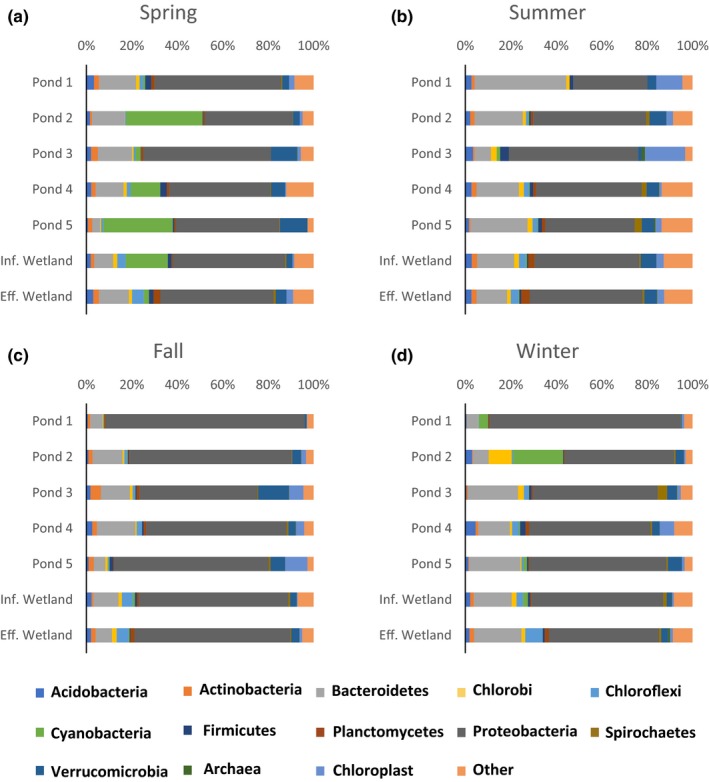
The relative abundance of 16s *rrn* sequences at the phyla level during four consecutive seasons in the remediation system at Wingfield Pines. The five remediation ponds, and the influent and effluent of the wetlands are shown

Using the 2,420,962 paired V4 *rrn* sequences, relative abundance at the phyla level showed that Proteobacteria were the most dominant phylum throughout the remediation system, regardless the time of year (Figure 3). They accounted for as high as 88% of all sequences. Fall (Figure 3C) and winter (Figure 3D) had the highest percentage of Proteobacteria, ranging from 48% to 88% of total bacterial phyla, while spring (Figure 3A) and summer (Figure 3B) had a greater diversity (as low as 33% Proteobacteria). Bacteroidetes and Cyanobacteria were the second and third largest phyla found, respectively. Bacteroidetes were found consistently throughout the year and ranged from as little as 3% (pond 5 in spring) to 40% (pond 1 in summer). Cyanobacteria were most prevalent in the spring, accounting for up to 34% of the relative abundance in pond 2. Collectively, these three phyla accounted for 65% to 93% of all bacterial *rrn* sequences found in a given sample.

**Figure 4:**
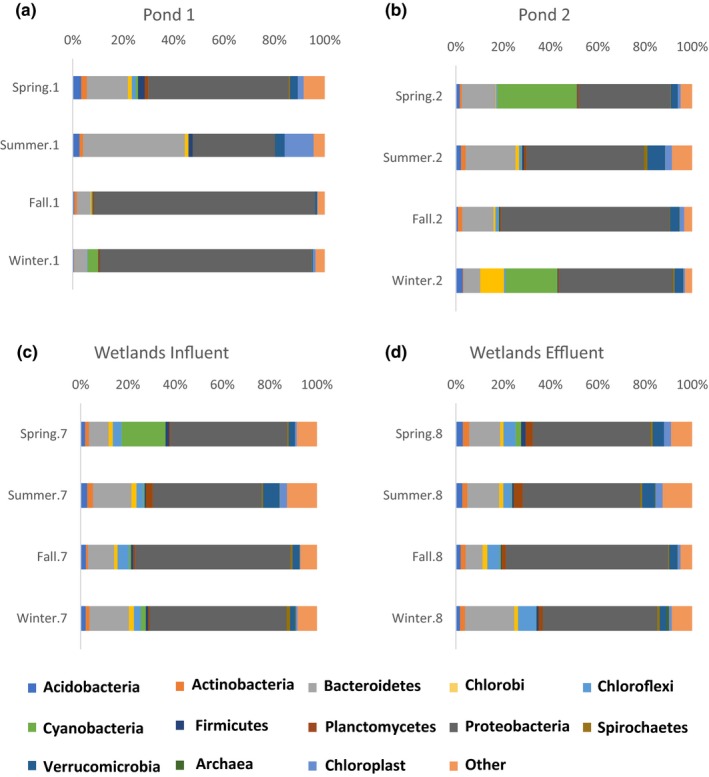
The relative abundance of 16s *rrn* sequences at the phyla level by geographic location. Panel A contains relative abundance for Pond 1; Panel B, Pond 2; Panel C influent to the wetlands; and Panel D, effluent from the wetlands and the system

The beginning of the remediation system exhibited the most variability in relative abundance when sequence data from the four seasons were compared (pond 1–2, Figure 4). In pond 1, Proteobacteria increased from 33% in the summer to 88% in the fall (Figure 4A). Pond 2 had significant amounts of Cyanobacteria in the spring and winter (34% and 22%) and few in the summer and fall (<1%; Figure 4B). At the end of the remediation system, more consistent, stable microbial communities were observed, with very similar phyla in the influent and effluent of the wetlands in each season (Figure 4C and D). One exception to this stable bacterial environment at the end of the remediation was the spike of Cyanobacteria sequences in the wetlands influent in the spring.

The Proteobacteria sequences were analyzed further to determine the relative abundance of the classes in this phylum (Figure S1). Betaproteobacteria were the most prevalent and were dispersed across the remediation system, accounting for as high as 60% of all bacteria. Deltaproteobacteria were the second most abundant and were distributed in a similar fashion as Betaproteobacteria*,* with the highest numbers found in pond 3 during the winter (45%; Figure S1D). Alphaproteobacteria occurred in high numbers in the spring (Figure S1A) and summer (Figure S1B), especially in pond 3 during the summer (29%). In contrast, there were <1% Alphaproteobacteria found in pond 3 during the winter (Figure S1D). Epsilonproteobacteria (<12%) were found primarily at the end of the remediation in pond 5, and the influent into and effluent from the wetlands.

Rarefaction plots were collated using phylogenetic distance (PD) whole tree, Heip's evenness, Chao1, and observed species richness metrics (Figure S2). The beginning of the remediation during the summer had very little species diversity (pond 3 <80 species), while pond 5, pond 6, influent and effluent of wetlands all had a sample richness over 1,000 species. Most of the samples ranged between 200 and 600 species, with a few closer to 800 species. Site 1 in the summer had <20 observed species due to low sequence depth (<1,000). Because quality filtering of sequences (deletion of sequences accounting for <0.005%) drastically reduced the sample size of pond 1 summer, an accurate representation could not be determined.

**Figure 5:**
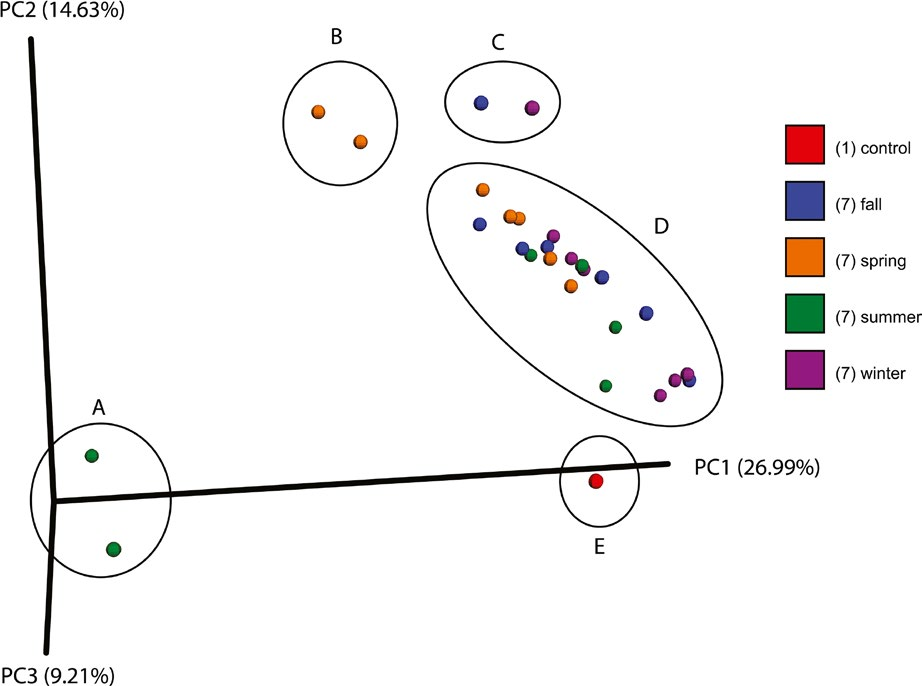
A principal‐coordinate analysis (PCoA) plot of all 29 samples (4 seasons at 7 sites and 1 control). Using Unifrac distances of samples, the PCoA plot shows 5 groups of samples. Bacterial communities from summer pond 1 and 3 (A) clustered, as did spring 2 and 3 (B), and fall 1 and winter 1 (C). The majority of the samples clustered into one large group (D). The control (E) was unique. Spring samples are represented by the orange dots, summer by green, fall by blue, and winter by purple

**Figure 6:**
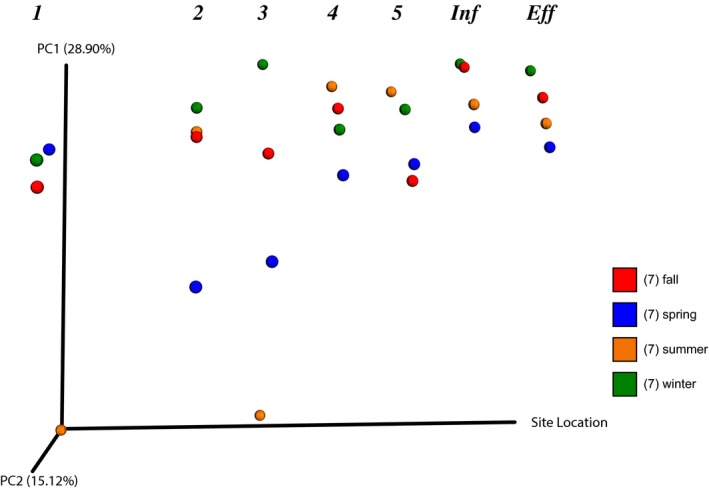
A PCoA plot with the sample site location as the *x* axis where spring samples are represented by blue dots, summer samples by orange dots, fall samples by red dots, and winter samples by green dots. The four dots (one of each color) under the 1 represent the communities in each of the 4 seasons in Pond 1; 2 is Pond 2, 3 is Pond 3; 4 is Pond 4; 5 is Pond 5, Inf is influent to the wetlands; and Eff is effluent from the system

When comparing data from the four seasons at each of the 8 collections sites, beta‐diversity analyses revealed that changes occurred throughout the remediation system. When graphed on a principal‐coordinate analysis (PCoA) plot, the samples clustered into five groups (Figure 5). Group A contained the summer samples from pond 1 and 3. This group is very different from all the others, indicating that the samples had unique bacterial communities that were evolutionary divergent from the rest of the samples. Group B contained spring samples (pond 2 and 3). Group C contained pond 1 for the fall and the winter. Group D contained most of the samples, showing that the bacterial composition across much of the remediation system was generally similar, despite some seasonal changes. Adonis reveals significant change by season (*r* ^2^ = .2566, *F* = 0.002) and to a lesser extent by site (*r* ^2^ = .11097, *F* = 0.003). When site location is set on the *x* axis of a PCoA plot (Figure 6), pond 3 shows the greatest difference in bacterial lineages throughout the year. Pond 1 in the summer and pond 2 in the spring were unique, and the rest of the remediation had a more uniform sequence composition.

A control was run using the MoBio soil kit and sterile nanopure water, as it has been reported that the soil kit reagents can show contamination in 16S *rrn* sequencing experiments (Salter et al., 2014). The control contained 4,268 sequences and 84 species, most of which were in trace amounts (<1%). It showed a different composition from the remediation site samples (Figure 5, Group E). Five species account for 69% of the 4,268 control sequences: *Acidobacteria* CCU21 (17%), *Chloroflexi* Ellin6529 (15%), *Deltaproteobacteria Desulfobacteraceae* (11%), *Deltaproteobacteria Desulfobulbaceae* (17%), and *Verrucomicrobiaceae* (9%). Of these, only *Desulfobacteraceae* and *Desulfobulbaceae* are found in the remediation site samples in significant abundance as well. For this reason, they are not considered significant in the analyses of the site samples.

When examined at the more specific taxonomic levels of families or genera, 9 groups accounted for 18% of the ~2.4 million sequences identified (Table S2). The family, *Comamonadaceae* sp*.,* had the highest (3.78%) relative abundance for the year, and was found in every sample except for pond 3 in the summer, which was the least diverse sample collected. *Comamonadaceae* sp. accounted for 25.96% of all bacteria found in pond 5 during the summer, which was more than double the second highest sample of 10.48% in pond 5 in the spring. With a relative abundance of 3.74%, the genus *Geobacter* was a close second. *Geobacter* were evenly dispersed throughout the remediation system but were not detected in pond 1 and 3 during the summer. *Phormidium* had the third highest number of sequences, with a yearly relative abundance of 3.32%. *Phormidium* were the highest in the spring in pond 4 (11.83%), pond 5 (29.01%), and the wetlands influent (17.93%). High levels were also found in pond 2 during the winter, where *Phormidium* accounted for 17.93% of all sequences. The sulfate‐reducing bacteria family *Desulfobulbaceae* was responsible for over 3% of the relative abundance (3.08%), however, it was also found in high abundance in the control. *Planktothrix, Rhodobacteracea* sp., *Gallionella*,* Dechloromonas*, and *Luteolibacter* all had a total relative abundance ~1.5%.

The major contaminants in Wingfield Pines are iron and sulfate. Iron was remediated in this study but sulfate passed through the system. We examined the prevalence of bacteria known to use iron and sulfate in their metabolism (Table 1). *rrn* sequences from iron‐reducing bacteria (IRBs) and iron oxidizing bacteria (IOBs) were most prevalent in the fall and winter. IOBs were most frequently found in Ponds 2 and the wetlands effluent. IRBs were most prevalent in Ponds 3, 4 and 5. The two groups of bacteria were not found in high numbers in the same ponds during the fall or winter. Sequences for sulfur/sulfide‐oxidizing bacteria (SOBs) and sulfur/sulfate‐reducing bacteria (SRBs) are also found in the largest numbers in the fall and winter. Both groups of bacteria are prevalent in ponds 4, 5 and the wetlands effluent.

**Table 1:** Elevated observed sequences of iron and sulfate cycling bacteria

Genus	Role^a^	SP^b^	SU^b^	FA^b^	WI^b^	Total Seq	Location^c^ 1	2	3	4	5	WI	WE
*Gallionella*	**IOB**	82	754	19,233	30,069	50,138		**F‐36%**					
								**W‐46%**					
*Dechloromonas*	**IOB**	3,182	850	33,061	10,907	48,000							**F‐60%**
*Geobacter*	*IRB*	4,178	3,594	30,849	99,291	137,912			*F‐15*%				
									*W‐10*%	*W‐42*%	*W‐11*%		
*Sulfuricurvum*	**SOB**	172	1,371	7,400	35,717	44,662					**W‐66%**		
*Rhodobacter*	**SOB**	1,662	747	5,283	7,694	15,386							**F‐10%**
										**W‐11%**	**W‐10%**		**W‐22%**
*Sulfuritalea*	**SOB**	253	560	6,375	5,742	12,930		**F‐17%**		**F‐14%**			
										**W‐19%**			
*Desulfobulbus*	*SRB*	1,804	828	9,041	4,673	16,346					*SP‐10*%		
										*F‐48*%			
*Desulfococcus*	*SRB*	677	760	2,700	10,021	14,158						*F‐10*%	
											*W‐27*%	*W‐14*%	*W‐14*%

IOB, iron‐oxidizing bacteria; IRB, iron‐reducing bacteria; SOB, sulfur/sulfide‐oxidizing bacteria; SRB, sulfur/sulfate‐reducing bacteria; SP, spring; SU, summer; F, fall; W, winter*;* 1–5, pond 1–pond 5; WI, wetland influent; WE, wetland effluent.

In the Role column, BOLD indicates oxidizing bacteria (IOB, SOB) and ITALIC indicates reducing bacteria (IRB, SRB).

Under the Location Columns, bold indicates the bacteria we specifically address in the text as potentially very important to the functioning of the remediation system and the geochemistry we measured.

a Predicted role based on published reports.

b Number of sequences for each genera by season.

c Values listed if >10%, % is the percentage of the total sequences for each genera found in each site, at a given season.

## 4 DISCUSSION

Pennsylvania contains 300 passive remediation systems for AMD and each system was designed to fit the AMD chemical profile, location and monetary constraints of the site. These 300 systems represent 300 independent experiments in remediating AMD. The purpose of this study was to examine the impacts of seasonal changes on the water quality and microbial communities of one of these passive remediation systems, with the long‐term goal of comparing different systems for bacterial communities, indicator bacteria, and contaminant remediation. The Wingfield Pines passive remediation system is comprised of an aeration pond (pond 1), 4 settling ponds (ponds 2–5), and a constructed wetland. Bacteria accumulated naturally in the system, without specific input.

We examined the levels of 12 metals and sulfate, and, using metagenomics, bacterial community composition, once per season, over the course of 1 year. Samples of water and soil slurries were taken at the entrance to each pond, and the influent and effluent of the wetland. Due to the circum‐neutral mine discharge, the pH remained neutral throughout the system, only increasing slightly from influent (pH 6.89) to effluent (pH 7.25). This was expected as passive systems often show an increase in pH during remediation (Hedin et al., 2013). Our data indicate that the remediation system can be divided into 3 unique environments. Ponds 1 and 2 had the highest levels of contaminants (Figure 2) and some seasonal variation in bacterial community composition (Figs. 5 and 6). Pond 3 had the most changes in contaminant levels, including seasonal metal spikes, and the highest seasonal variation in bacterial community composition of any part of the remediation system (Figs. 2 and 6). Ponds 4, 5, and the wetlands were very similar in contaminant levels and in seasonal bacterial community composition. The wetlands had some seasonal spikes in specific metals (Figs. 2 and 6).

Passive remediation systems for AMD are mainly concerned with oxidation‐reduction reactions. These reactions can be either abiotic or biotic and can be influenced by oxic and anoxic conditions. For example, aeration of neutral pH AMD containing large concentrations of iron results in rapid, abiotic precipitation of iron oxide, while specific bacteria, through their metabolic reactions, can also influence the redox state of the iron. Many different genera of iron‐reducing bacteria (IRBs) and iron‐oxidizing bacteria (IOBs) have been described. IOBs can contribute to iron oxidation in circum‐neutral environments by residing at the oxic‐anoxic zone near the bottom of the ponds (Hedrich, Schlömann, & Johnson, 2011). It is known that some precipitates sequester other heavy metals. For example, both iron oxyhydroxides (Bourg & Loch, 1995) and manganese oxyhydroxides (Boonfueng, Axe, Xu, & Tyson, 2006) are capable of sequestering heavy metals in precipitates using abiotic reactions. Reduction in these oxyhyroxides can result in resolubilization and remobilization of the metals (Davranche & Bollinger, 2000; Kappler & Straub, 2005; Quantin, Becquer, Rouiller, & Berthelin, 2001; Shope et al., 2006). Cyanobacteria are capable of sequestering heavy metals as biomass using a number of strategies. Heavy metals bind to and are trapped by the exopolysaccharides of the cells. It is estimated that a third to a half of cytoplasmic enzymes have metal cofactors, with many of the enzymes of photosynthesis using metals in their active sites (Cassier‐Chauvat & Chauvat, 2014). Our last concern in these studies was the geographic locations of the contaminants and the bacteria. For example, abiotic iron precipitation results in precipitates that settle on or near the bottom of the ponds. The system is designed so that the precipitates stay in the ponds and the decontaminated water flows through the system. It is well documented that reducing bacteria are mainly anaerobes and their growth would be supported in the suboxic and anoxic zones near or on the bottom of the ponds, in the same geographic location of the iron precipitates. Thus, it is possible that reducing bacteria could resolubilize the precipitates and remobilize the metals.

The passive remediation system at Wingfield Pines was designed to remove large quantities of dissolved iron from AMD and our data indicated that the system was efficient in iron removal for most of the year (81%), with a decline in iron removal in the fall (51%). Water quality analyses revealed a decrease in iron entering the system in the winter (Figure 2), which is consistent with other reports of AMD in Pennsylvania (Hedin, 2008). Thus, we observed a seasonal effect on iron concentration and remediation in Wingfield Pines. During the fall, there was a significant amount of vegetation decomposition in the wetlands of Wingfield Pines, leading to both an increase in nutrients for bacteria and anoxic conditions in more of the wetlands. An examination of iron cycling bacterial sequences (IOBs and IRBs; Table 1) revealed a striking pattern. Both groups of bacteria increased dramatically in the fall and winter (Table 1, columns 5 and 6), but in different locations in the system (Table 1, columns 8–14). While the sequences were found throughout the system, IOBs, which would aid in precipitating iron, were mainly found in pond 2 (fall and winter) and the wetlands effluent (fall), whereas IRB sequences from bacteria that could resolubilize iron precipitates were found in greater number in the system and mainly in ponds 3, 4 and 5. The location of these bacteria, coupled with the amount of iron entering the system and decomposition levels in the wetlands may explain the decreased capacity of the system for iron remediation in the fall. The fact that there was less iron entering the system in the winter and it could be remediated under similar conditions as the fall, suggests that this lower level of iron can be remediated in the system.

Spikes in aluminum, copper, lead, nickel, manganese and zinc were seen in Pond 3 during the summer and the wetlands during the fall. Additionally, these metals were present in very low concentrations at the beginning of the system and gradually increased, with the highest levels occurring in the effluent. For Al and Mn, this trend was seen during all seasons but was the most pronounced in the fall. The increase in concentration from influent to effluent was unexpected. In the case of aluminum, abiotic solubilization requires an acidic pH of 5 or lower (Hedin et al., 1994). Because the pH in the system is neutral, this suggests a biotic role for the aluminum spikes. It has been well documented that both iron and manganese oxyhydroxides, which are very common in AMD, can trap heavy metals, including aluminum, manganese, and nickel. Aluminum is the most mobile in these precipitates (Rose & Ghazi, 1998). *Geobacter,* one of the most well‐studied IRBs, also have the ability to reduce manganese (Lovley & Phillips, 1988; Lovley, Stolz, Nord, & Phillips, 1987). *Geobacter* is the second most prevalent genus of bacteria in the system (Table S2). It is likely that the reduction in iron oxyhdroxides are the main drivers of resolubilization at the beginning of the system (as seen in pond 3), while reduction in manganese oxydroxides are responsible for the resolubilization in the wetlands. This conclusion was drawn because the wetlands exhibit a noticeable increase in manganese in the fall where the metal contamination spikes are observed, while the iron does not follow the same trend (i.e., remains low throughout the wetlands).

The sulfur cycle includes the oxidation of reduced sulfur compounds to sulfate (and sulfur intermediates), as well as sulfate reduction to sulfide, which can then form metal sulfides. Sulfate is predominant in AMD (Capo et al., 2001; Hedin et al., 1994, 2013), indicating that successful removal of sulfate requires reduction to sulfide and precipitation of metal sulfides. *Desulfococcus* and *Desulfobulbus* are the dominant sequences of SRBs and are found primarily in the fall and winter (Table 1). SOB sequences were twice as prevalent as SRBs and dominated by two genera, *Sulfuricurvum* and *Sulfuritalea*. These genera were also found predominately in the fall and winter (Table 1). Despite the presence of both SOBs and SRBs, sulfate levels remained constant and unremediated. It is possible that the ratio of SRBs:SOBs is important for sulfate remediation. Likewise, the total number of SRBs present could influence the remediation.

Sulfate levels were constant throughout the four testing periods and high enough to create an environment for anaerobic microbes to use sulfate as an electron acceptor under anaerobic conditions (Hallberg, 2010). This could explain the soluble barium that was observed in the system (Figure 2), as it has been found that sulfate‐reducing bacteria often use barite (BaSO_4_) as a sulfate source in anaerobic respiration (Bolze, Malone, & Smith, 1974; McCready & Krouse, 1980). Barite is likely the form that barium would take in the remediation site due to the elevated levels of sulfate and it is typically insoluble in water. Though sulfate‐reducing bacteria favor ferrous sulfate over barite, it has been found that in a mixed environment (barite and ferrous sulfate), barite is still utilized resulting in low levels of soluble barium (Bolze et al., 1974).

Ponds 1 and 2 have the highest variability in relative abundance of bacteria, whereas the wetlands have the most stable and most diverse bacterial communities (Figure 4). PCoA plots indicated that 3 outlying clusters of bacteria represented samples from ponds 1, 2 or 3 (Figure 5). Pond 1 and 3 in the summer (Figure 5A) clustered tightly together and far from the other samples. The clustering can be explained due to a minimal number of observed species (Figure S2), few iron or sulfur (oxidizing or reducing) bacteria (Table 1) and the largest amount of chloroplast sequences (Figure 3). The clustering of ponds 2 and 3 in the spring (Figure 5B) can be explained by few iron and sulfur bacteria (Table 1) and a minimal number of chloroplast sequences (Figure 3). The clustering of pond 1 in the fall and winter can be explained by the presence of one iron‐oxidizing bacteria, *Gallionella,* where its observed sequences increased by sixfold in these two sites (Table 1).

The relative abundance of sequences at the phyla level showed that Proteobacteria were the most common bacterial sequences across the remediation system (33%–88% of a given sample; Figure 3). This is to be expected as most of the iron and sulfur‐oxidizing and reducing bacteria are found in this phyla (Emerson, Fleming, & McBeth, 2010; Fike, Bradley, & Rose, 2015; Hedrich et al., 2011; Scala, Hacherl, Cowan, Young, & Kosson, 2006). Rhodobacteraceae*,* one of the most abundant families in Wingfield Pines (Table S2), is an Alphaproteobacteria*. Rhodobacter* is known for both iron and sulfur oxidation (Fike et al., 2015; Hedrich et al., 2011) and *Rhodobacter* spp. sequences were found throughout the remediation system with the highest numbers in pond 5 and the effluent in the fall and winter. Betaproteobacteria fluctuated throughout the year, but were the most prevalent in pond 1 during the fall (54% total site composition) and winter (60% total site composition). This appears to be a direct correlation with the significant increase in the Betaproteobacteria, *Gallionella,* in pond 1 in the fall and winter (Table 1). Comamonadaceae spp. are Betaproteobacteria consisting primarily of bacteria that are capable of both denitrification and Fe(III) reduction (Willems, 2014) and the highest observed sequence counts were found in pond 5 during the spring and fall (Table S2). *Dechloromonas,* known for the ability to use (per)chlorate as an electron acceptor, is also capable of oxidizing iron in anaerobic conditions by respiring on nitrate (Weber, Achenbach, & Coates, 2006). Sixty percent of all *Dechloromonas* sequences were found in the wetland effluent during the fall. It is most probable that *Dechloromonas* spp. are anaerobically oxidizing iron in the wetlands during the fall where iron levels are highest (Table S2, Figure 2), and when plant death and N cycling are high. Deltaproteobacteria were present in all samples in varying amounts (2%–45%) and identified primarily sulfate‐reducing bacteria. The largest group of SRB sequences found belonged to *Desulfococcus, Desulfobulbus, Desulfobacca,* and *Desulfomonile*. There were very few Epsilonproteobacteria present in the system, they were found toward the end of the remediation, and the class was dominated by the *Sulfuricurvum* (1% all sequences) which contain known sulfur‐oxidizing bacteria (Handley et al., 2014; Kodama & Watanabe, 2004). Interestingly, of the 44,662 observed sequences for *Sulfuricurvum* spp., 29, 482 of the sequences were found in pond 5 in the winter. Pond 5 in the winter had an increase in both sulfur‐oxidizing and reducing bacteria (up 10% from the average of 4%) and iron‐oxidizing and reducing bacteria (up 4% from average of 6%). The Gammaproteobacteria present represented a more diverse group of bacteria, containing bacteria indigenous to soil and freshwater aquatic environments (Šimek et al., 2005).

We observed large mats of photosynthetic organisms that visibly increased in biomass at the surface of the ponds during the spring and summer (Figure S3). Concurrently, Cyanobacteria sequences increased in number. Field measurements of dissolved O_2_ indicated that pond 3 had double the dissolved O_2_ in the mats of photosynthetic organisms (~18 mg/L) as compared to other ponds (~9 mg/L) and very low concentrations of dissolved O_2_ immediately below the mats (data not shown). We hypothesize that these two observations may result in inverse effects on remediation. Cyanobacteria can decrease heavy metal contaminant levels within the mats, while metal‐reducing bacteria, which are largely anaerobic organisms (Caccavo et al., 1994; Lovley, 2002) could grow in the anoxic zones underneath the photosynthetic mats and resolubilize contaminants.

Our data indicate the potential impacts of bacterial communities on AMD remediation in passive systems. From these data, we have generated a series of testable hypothesis to examine these potential impacts in detail. By comparing the data from Wingfield Pines to other AMD remediation systems in PA, we will be able to identify indicator bacterial communities and inform the efficacy and efficiency of these passive systems.

## CONFLICT OF INTEREST

None declared.
