# Supplementary material for: A seasonal study of a passive abandoned coalmine drainage remediation system reveals three distinct zones of contaminant levels and microbial communities
Source: Microbiologyopen. 2018 Apr 25;7(4):e00585. doi: 10.1002/mbo3.585 (PMC6079175; doi:10.1002/mbo3.585)
Supplement: Supplementary file 1 [file MBO3-7-e00585-s001.pdf]

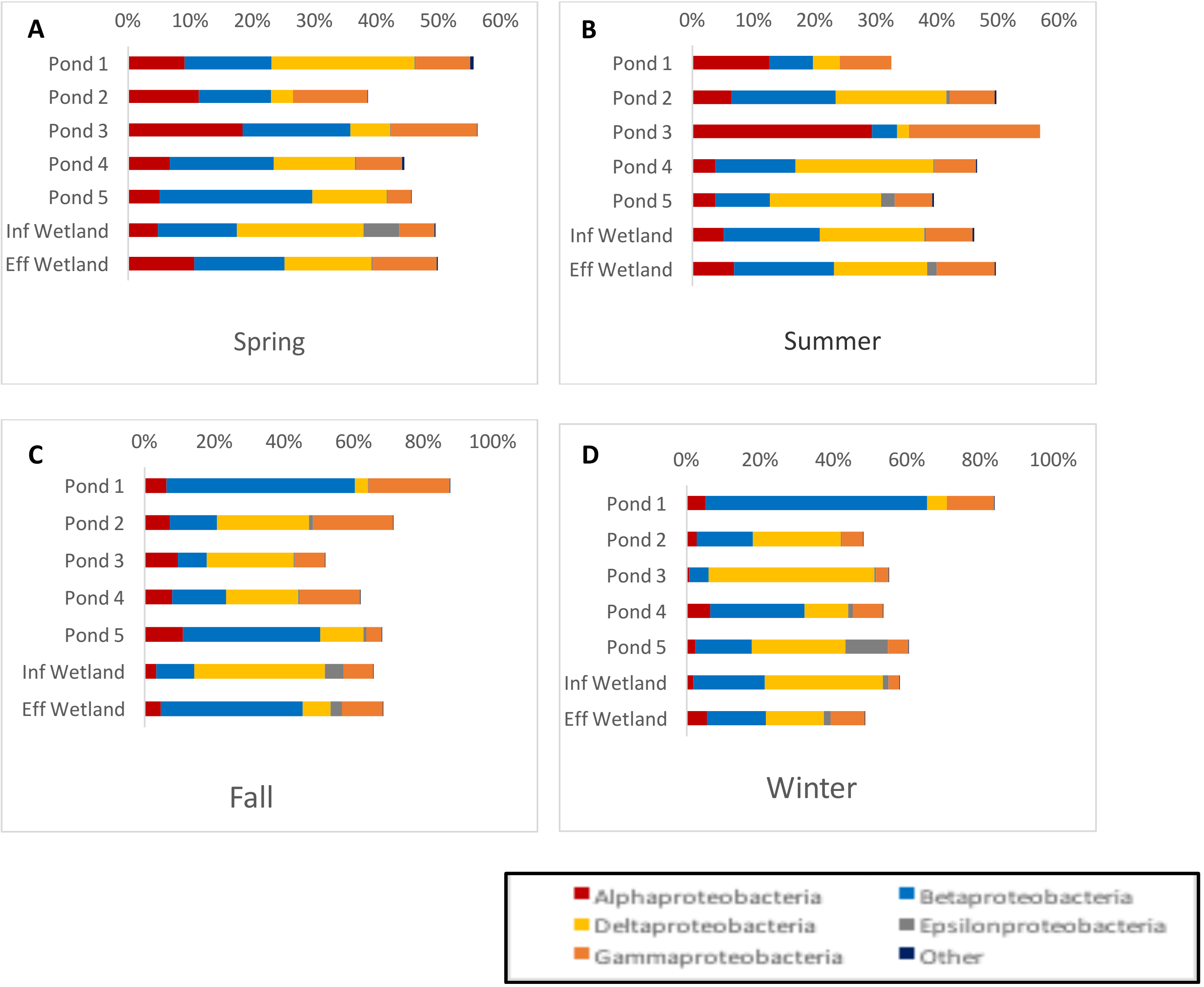

Fig. S1. The relative abundance of classes found in the most prevalent phylum, Proteobacteria. Alphaproteobacteria were much higher in the spring and summer than in the fall and winter. Betaproteobacteria were most prevalent across the entire remediation system. Epsilonproteobacteria were the least prevalent (<12%) and primarily at the end of the remediation system. The percentage do not total to 100% because phyla other than Proteobacteria make up the difference.

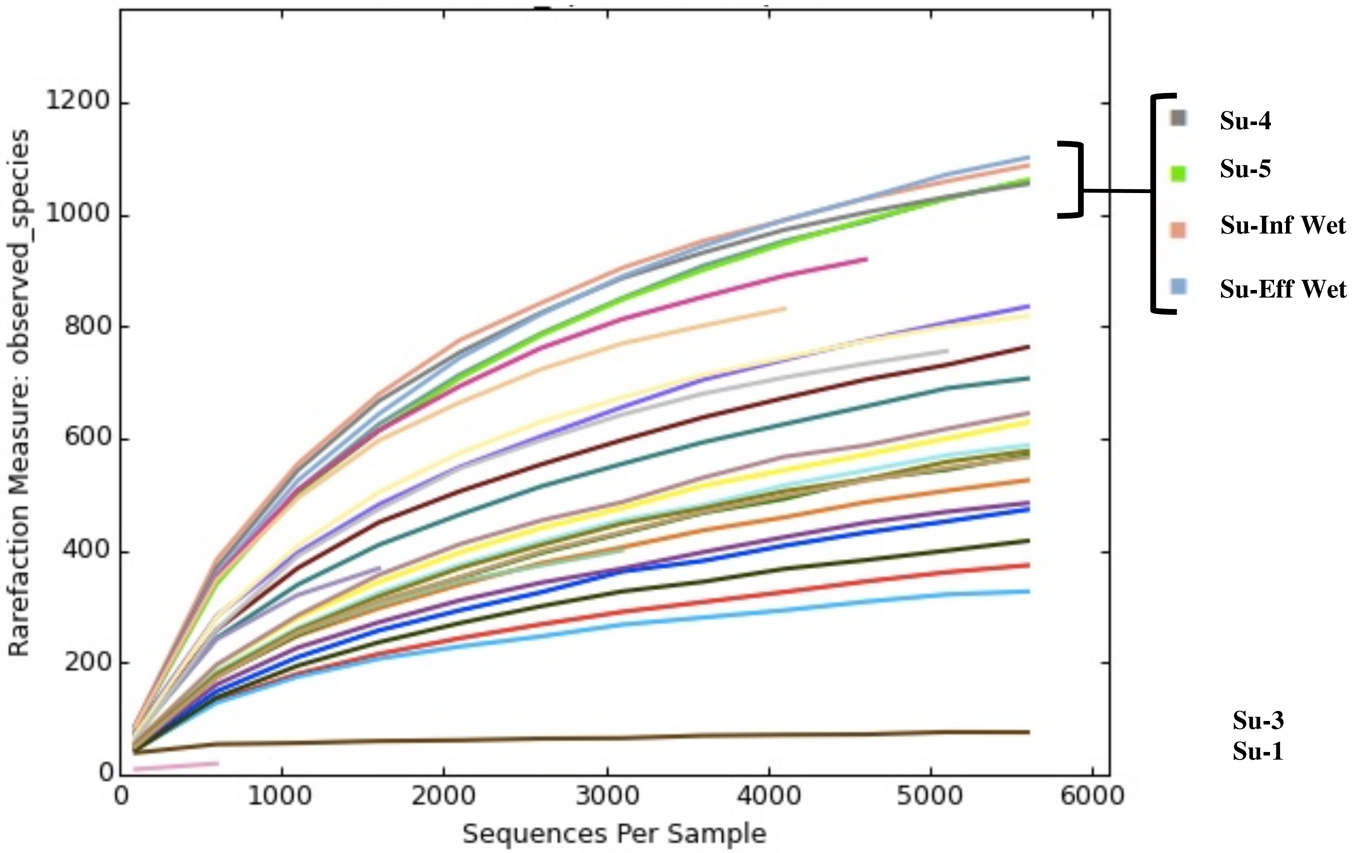

Fig. S2. The alpha diversity of observed species shows that summer has the largest range in diversity in bacterial communities, having both the most (4, 5, Inf Wet and Eff Wet) and least (1 and 3) rich samples. Pond 4 through the effluent had the highest number of observed species (>1000), while pond 3 had the least (<80). Site 1 in the summer had <20 observed species but due to a low number sequences (after removal of sequences accounting for <0.005%) it cannot be determined if that is an accurate representation of sample diversity.

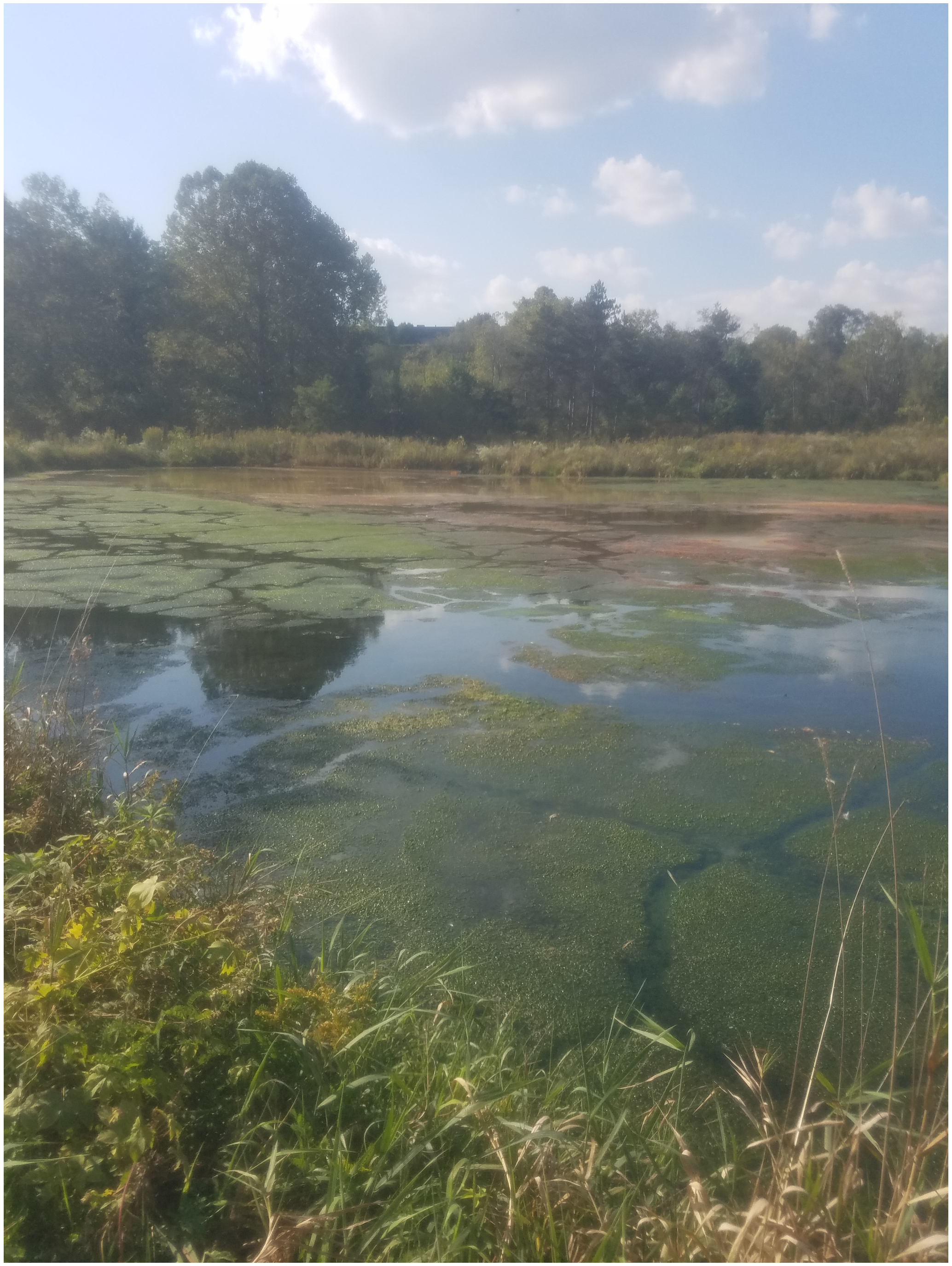

Fig. S3 Photosynthetic mats in Pond 3 of the Wingfield Pines Passive Remediation System during the summer.

Table S1: Water quality report of yearly averages by site using the seasonal data

all values in mg/L	aluminum	barium	copper	iron	lead	manganese	nickel	strontium	sulfate	zinc	pH
Pond 1	0.10	0.10	<0.01	213.23	<0.03	0.43	<0.01	3.11	292.50	0.03	6.89
Pond 2	1.86	0.07	<0.01	90.13	<0.03	0.99	<0.01	2.87	287.00	0.04	6.97
Pond 3	5.48	0.02	0.02	75.05	0.04	1.96	0.02	2.86	292.50	0.08	7.16
Pond 4	2.28	0.08	<0.01	87.35	<0.03	1.20	<0.01	2.68	288.25	0.06	7.11
Pond 5	1.03	0.08	<0.01	62.93	<0.03	1.77	<0.01	2.79	285.00	0.04	7.16
Inf Wetlands	1.48	0.05	<0.01	58.75	<0.03	2.69	<0.01	2.76	291.75	0.03	7.15
Eff Wetlands	5.08	0.09	0.02	41.27	0.07	2.78	0.03	3.12	298.25	0.10	7.25

Table S2: The relative abundance of the 9 most prevalent genera found across the remediation

Genus	Elevated Values*	Total Relative Abundance (%)
Comamonadaceae sp.	SP-3 (6.22%),	3.78%
	SP-5 (10.48%)	
	F-5 (25.96%)	
Geobacter	SP-1 (5.53%)	3.74%
	F-2(18.9%)	
	W-2 (7.28%)	
	W-3 (27.50%)	
	W-5 (5.77%)	
Phormidium	SP-4 (11.83%)	3.32%
	SP-5 (29.01%)	
	SP-WI (17.93%)	
	W-2 (21.70%)	
Desulfobulbaceae sp.	SP-WI (8.52%)	3.08%
	F-WI (14.21%)	
	W-2 (7.46%)	
	W-3 (7.92%)	
	W-WI (7.85%)	
Luteolibacter	SP-3 (7.80%)	1.67%
	SP-5 (9.92%)	
	F-3 (5.18%)	
Dechloromonas	F-WE (22.81%)	1.56%
Gallionella	F-1 (15.59%)	1.35%
	W-1 (13.92%)	
Rhodobacteraceae sp.	SU-1 (6.13%)	1.32%
	SU-3 (12.84%)	
Planktothrix	SP-2 (27.23%)	1.27%

** Values listed if >5%. SP = Spring, SU = Summer, F = Fall, W = Winter,* WI = Wetland Influent, WE = Wetland Effluent, 1-5 = Pond 1 - Pond 5.
